# Occupational and Physical Therapy Strategies for the Rehabilitation of COVID-19-Related Guillain-Barré Syndrome in the Long-term Acute Care Hospital Setting: Case Report

**DOI:** 10.2196/30794

**Published:** 2022-02-10

**Authors:** Catherine Connors, Stephanie McNeill, Henry Charles Hrdlicka

**Affiliations:** 1 Department of Inpatient Physical Therapy Gaylord Specialty Healthcare Wallingford, CT United States; 2 Department of Inpatient Occupational Therapy Gaylord Specialty Healthcare Wallingford, CT United States; 3 Milne Institute for Healthcare Innovation Gaylord Specialty Healthcare Wallingford, CT United States

**Keywords:** Gullian-Barre syndrome, COVID-19, SARS-CoV-2, occupational therapy, physical therapy, long-term acute care hospital, rehabilitation, case report, treatment, diagnosis

## Abstract

**Background:**

Although several reports have described the diagnosis and treatment of patients with COVID-19-associated Guillain-Barré syndrome (GBS), there is a paucity of literature describing the occupational and physical therapy (OT and PT) strategies used in the long-term acute care hospital (LTACH) setting to rehabilitate these patients.

**Objective:**

To expand this body of literature, we present a case report highlighting the treatment strategies used to rehabilitate and discharge an individual from an independent LTACH facility, following diagnosis and treatment of COVID-19-related GBS at a regional ACH.

**Methods:**

A 61-year-old male was admitted to an LTACH for the rehabilitation of GBS following COVID-19 infection and intravenous immunoglobulin treatment. Rehabilitation in the LTACH setting uses a variety of skilled treatment interventions to meet patient-driven goals and maximize their function to the highest level possible in preparation of their discharge to a subacute or homecare setting. In this case, this was accomplished through individual OT and PT sessions, OT/PT cotreatment sessions, and targeted group therapy sessions focused on leg, arm, and fine motor coordination exercises.

**Results:**

With the OT and PT standard of care, the patient’s improvement was demonstrated by several outcome measures, including manual muscle testing, range of motion, grip strength, and the activity measure for postacute care. The patient was successfully rehabilitated and returned to the community after presenting with COVID-19-associated GBS.

**Conclusions:**

This report highlights the complex rehabilitation needs patients require to regain independence after diagnosis of COVID-19-associated GBS.

## Introduction

### Background

SARS-CoV-2 is a novel coronavirus strain that has led to the emergence of the COVID-19 pandemic [1] and over 5.3 million deaths as a result [2]. As with other infectious vectors, including coronavirus strains SARS-CoV and Middle East respiratory syndrome (MERS) [3-7], the immune response elicited by SARS-CoV-2 has been implicated in the etiology of several neurological disorders, such as stroke, and autoimmune diseases, including Guillain-Barré syndrome (GBS) [5-11]. GBS is exceedingly rare, affecting 1-2 of every 100,000 people in the United States, or 0.001%-0.002% [12]; in contrast, a 0.42% incidence rate of GBS in individuals diagnosed with COVID-19 has been reported [10,13].

GBS, an acute autoimmune polyradiculopathy disorder characterized by symmetrical progressive ascending weakness, areflexia, and sensory loss closely resembling quadriplegia, is typically the result of molecular mimicry and the formation of autoantibodies targeting the proteoglycans common to the myelin sheath [3,4]; the specific autoantigen linked to SARS-CoV-2 infection and GBS is still under investigation [7,9].

In rare cases, GBS can lead to irregular heart rhythms, respiratory distress, heart attacks, or death; the GBS mortality rate ranges from 3% to 10% of infections [3,14,15]. Respiratory insufficiency further complicates COVID-19 and GBS outcomes, as both diagnoses can cause respiratory distress and shortness of breath. As symptoms overlap, it is advisable for clinicians to be aware of the less-common symptoms of GBS, including diplopia and paresthesia, so appropriate treatments can be initiated in a timely manner [10,16]. Given that GBS is diagnosed and treatment started early to avoid serious cardiac and pulmonary complications, and there are no serious secondary infections, patients typically recover well, with 60%-80 % walking after 6 months [14].

Although many reports have documented the diagnosis and treatment of GBS following COVID-19 [17-32], the literature documenting the rehabilitation process of these individuals is limited. At the time of writing, only 1 such paper exists to the best of our knowledge [33]. In that paper, the patient, having been treated for GBS associated with cerebral vasculitis, was admitted to the rehabilitation unit of the same acute care hospital (ACH) for further care and rehabilitation. To expand this body of literature, we present this case report highlighting the treatment strategies used to rehabilitate and discharge an individual from an independent long-term acute care hospital (LTACH) facility, following diagnosis and treatment of COVID-19-related GBS at a regional ACH.

The treatment strategies described here are informed by the LTACH’s long history of treating patients with GBS. For example, between March 2019 and the start of March 2020, approximately 18 patients were treated for GBS at this facility. Since the start of the COVID-19 pandemic (March 2020-March 2021), this facility has treated 23 patients for GBS, 5 (21%) of which, including the case described here, were COVID-19-associated GBS cases.

### Case Presentation

**Figure 1:**
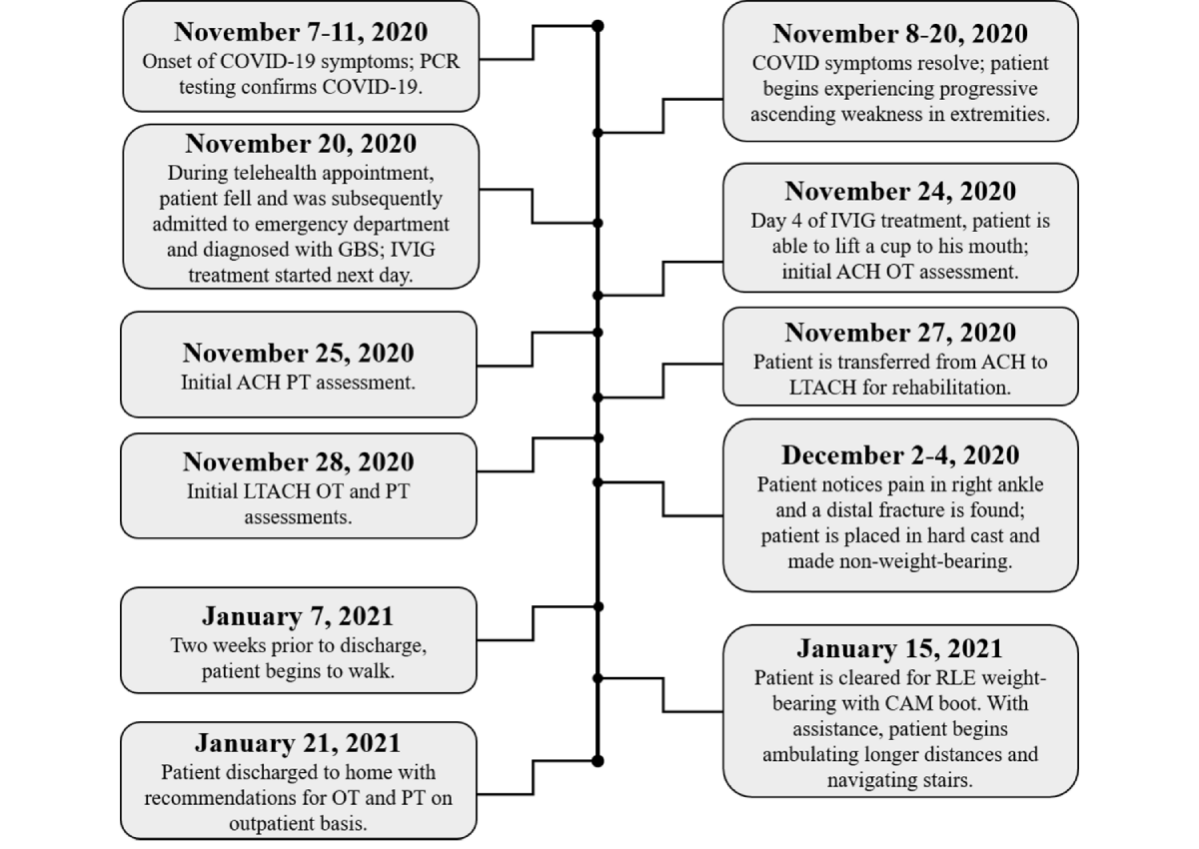
Patient timeline. Starting November 7, 2020, the timing of the patient’s diagnosis, treatment, rehabilitation, and other significant events are outlined until his discharge from the LTACH rehabilitation setting on January 21, 2021. ACH: acute care hospital; CAM: controlled ankle motion; GBS: Guillain-Barré syndrome; IVIG: intravenous immunoglobulin; LTACH: long-term acute care hospital; OT: occupational therapy; PCR: polymerase chain reaction; PT: physical therapy; RLE: right lower extremity.

On November 11, 2020, a 61-year-old Caucasian male tested positive for COVID-19 by polymerase chain reaction (PCR) testing, after developing shortness of breath and a low-grade fever on November 7, 2020 (Figure 1). By the time the patient received his test results, he was afebrile and his shortness of breath had begun to markedly improve. The patient then began developing progressive ascending weakness and numbness in both his upper and lower extremities (UEs and LEs). The weakness progressed to the point where it was increasingly difficult to ambulate and negotiate stairs; at this time, the patient began using a walker to assist with mobility.

Other than a history of hypertension and hyperlipidemia, the patient’s past medical history was unremarkable prior to the diagnosis of COVID-19 infection. The patient’s father is alive at 91 years old, and his mother passed away at 88 years old with a history of hypertension. He lives with his wife, who is a full-time caregiver to her mother, while the patient works full-time as an office shop manager. Prior to COVID-19 infection, he was independent with all activities of daily living (ADLs) and mobility.

On November 20, 2020, in lieu of an office visit due to state COVID-19 restrictions, the patient attended a telehealth appointment to discuss his symptoms. During the telehealth appointment, the patient fell when attempting to stand and was unable to get off the floor. Consequently, emergency medical services were called and the patient was brought to the emergency department under droplet precautions. At admission, the patient was awake and alert, with clear fluent speech and no facial asymmetry. His vital measurements were as follows: temperature, 36.9°C; blood pressure, 164/91 mm Hg; pulse, 105 beats per minute, oxygen saturation, 97% on room air; and respiratory rate, 18 breaths per minute. All laboratory tests collected were within normal ranges, including the following: white blood cell count, 7.8×10^3^ cells/μL; hemoglobin, 15.4 g/dL; platelet count, 299×10^3^ cells/μL; blood urea nitrogen, 9 mg/dL; creatinine, 0.6 mg/dL; aspartate aminotransferase, 21 IU/L; alanine aminotransferase, 34 IU/L; and albumin, 4.5 g/L. Upon physical examination, the patient presented with rapidly progressing UE and LE weakness with absent patellar and bicep reflexes.

Magnetic resonance imaging (MRI) of the brain ruled out acute infarction. Cervical MRI showed mild degenerative joint disease with disc desiccation from C2 to C7 discs without cord compression. A lumbar puncture was attempted on November 21, 2020, but was unsuccessful. The patient was ultimately diagnosed with postinfectious COVID-19-associated acute inflammatory demyelinating polyradiculoneuropathy (AIDP), a common GBS variant [10]. Once diagnosed, the patient was given a 5-day intravenous immunoglobulin (IVIG) cycle at 0.4 g/kg/day (November 21-26). On the fourth day of IVIG treatment, the patient was able to lift a cup to his mouth, which he was previously unable to do. After completing the day 5 IVIG treatment of the cycle, the patient presented with improving weakness in both UEs and LEs, while continuing to report pain in his UEs and tingling in both UEs and LEs. His positive response to IVIG treatment reinforced the GBS diagnosis.

Speech therapy was not requested at the ACH, as cognitive, swallowing, and communication deficits were not noted at that time. Additionally, since the progressive ascending weakness from GBS did not affect his respiratory system, and the shortness of breath secondary to COVID-19 had resolved prior to being admitted to the ACH, he did not require evaluation by respiratory therapy.

At the ACH, the patient’s arm strength was assessed by occupational therapy (OT) using manual muscle testing (MMT) and was as follows [34] (Table 1):

- Right upper extremity (RUE): shoulder 2/5, elbow 3–/5, and hand grasp 3/5
- Left upper extremity (LUE): shoulder 2–/5, elbow 2+/5, and hand grasp 2/5.

With regard to ADLs, the patient required built-up utensils and setup assistance to perform self-feeding and moderate assistance for oral care. Therapists at the ACH attempted to stand the patient; however, he was unable to achieve a fully upright position. As a result, he required the use of a mechanical lift for out-of-bed transfers.

On November 27, 2020, 7 days after admission, the patient was discharged to an LTACH, Gaylord Specialty Healthcare (Wallingford, CT, USA), for inpatient rehabilitation. His goal upon admission was to return to an independent level of function.

**Table 1:** Occupational therapy upper extremity assessments.

Assessment		T0^a^		T1^b^		T2^c^
**BUE^d^ strength**						
	ROM^e^	WNL^f^	WNL		WNL	
**RUE^g^ strength**						
	Shoulder	2/5	3+/5		5/5	
	Elbow^h^	3–/5	3+/5		5/5	
	Wrist^h^	—^i^	3+/5		5/5	
	Grip	—	15 lb		52 lb	
**LUE^j^ strength**						
	Shoulder	2–/5	3+/5		5/5	
	Elbow^h^	2+/5	3+/5		5/5	
	Wrist^h^	—	3+/5		5/5	
	Grip	—	21 lb		70 lb	
**ADLs^k,l^**						
	Self-feeding, oral care	ModA^m^	D^n^		I^o^	
	UE^p^ bathing, dressing	D	MinA^q^		DS^r^	
	LE^s^ bathing, dressing	D	MaxA^t^		CG^u^	
	Toilet and shower transfers	D	MaxA		S^v^	
**AM-PAC^w^** **OT^x^**		—		17 points; 50% impairment		20 points; 35% impairment

^a^T0: acute care hospital (ACH) OT admission assessment, November 24, 2020.

^b^T1: long-term acute care hospital (LTACH) OT admission assessment, November 28, 2020.

^c^T2: LTACH OT discharge assessment, January 20, 2021.

^d^BUE: bilateral upper extremity.

^e^ROM: range of motion.

^f^WNL: within normal limits.

^g^RUE: right upper extremity.

^h^Measurement of both flexion and extension.

^i^Not assessed at this time.

^j^LUE: left upper extremity.

^k^ADL: activity of daily living.

^l^ADL measurements based on a modified functional independence measure [35].

^m^ModA: moderate assistance required.

^n^D: dependent.

^o^I: independent.

^p^UE: upper extremity.

^q^MinA: minimal assistance required.

^r^DS: distant supervision required.

^s^LE: lower extremity.

^t^MaxA: maximal assistance required.

^u^CG: contact guard assistance required.

^v^S: supervision required.

^w^AM-PAC: activity measure for postacute care.

^x^OT: occupational therapy.

### Initial Functional Status at the LTACH

Upon initial evaluation by LTACH physical therapy (PT), the patient’s LE range of motion (ROM) was within normal limits (WNL), and his LE strength, assessed by MMT, was as follows (Table 2):

- Right lower extremity (RLE): hip flexion 3/5, hip abduction/adduction 2+/5, knee flexion 4/5, knee extension 3+/5, and ankle dorsiflexion 3/5
- Left lower extremity (LLE): hip flexion 3/5, hip abduction/adduction 2/5, knee flexion/extension 3/5, and ankle dorsiflexion 3–/5

Proprioception to bilateral great toes and ankles was absent, and the patient had diminished sensation to light touch in bilateral lower extremities (BLEs).

The patient’s functional status was evaluated using a modified functional independence measure ranging from dependent to independent (Table 3) [35]. He required maximal assistance to perform bed mobility and out-of-bed transfers. He also required contact guard (CG) assistance to maintain sitting balance on the edge of the bed. The activity measure for postacute care (AM-PAC), a standardized assessment tool, was used to measure the patient’s ability to complete ADLs and functional mobility [36]. The patient scored a 10 on the mobility segment during the initial PT evaluation, indicating 77% impairment (Table 2).

On initial evaluation by LTACH OT, the patient’s UE passive ROM was WNL and his bilateral upper extremity (BUE) strength, assessed by MMT, was as follows: (both RUE and LUE) shoulder flexion, 3+/5; elbow flexion/extension, 3+/5; and wrist flexion/extension, 3+/5. By a dynamometer, the patient’s right grip strength was 15 lb and 21 lb on the left (Table 1). His UE sensation was intact to light touch and deep pressure and had intact proprioception. However, he continued to endorse numbness and tingling in his hands. His coordination demonstrated dysmetria, as evidenced by decreased accuracy when performing a finger-to-nose assessment with occluded vision. The patient scored a 17 on the initial OT ADL portion of the AM-PAC evaluation, indicating 50% impairment (Table 1).

Upon admission to the LTACH, other than assistance to cut foods and open containers, the patient had progressed to requiring distant supervision for self-feeding and no longer required the use of built-up handles. Additionally, he required minimal assistance for upper body bathing and maximal assistance for lower body bathing at bed level. He required maximal assistance for upper body dressing and total assistance for lower body dressing at bed level.

All patients evaluated by OT at this LTACH facility are given the St. Louis University Mental Status (SLUMS) examination at admission to screen for possible cognitive impairments and to inform the treatment plan [37]. The patient scored a 25/30 on the SLUMS examination, indicating mild neurocognitive impairments in attention and short-term memory. The patient stated that his attention was a baseline impairment likely present prior to his GBS diagnosis but that his short-term memory was currently worse than his baseline status prior to hospitalization. Furthermore, the patient complained of blurry vision and difficulty reading since the onset of GBS. A formal vision assessment, performed by OT, confirmed blurriness in both eyes (right worse than left). The patient’s near point of convergence was 12 inches, indicating convergence insufficiency and a marked impairment compared to the normal range, which is between 2 and 4 inches. Clinically, this observation is intriguing as ocular muscle weakness and paralysis are associated with the Miller Fisher syndrome variant of GBS, not the AIDP variant [38].

During an initial OT/PT cotreatment session to perform standing in the parallel bars on December 2, 2020, 5 days after LTACH admission, the patient’s LE sensation began to improve and he complained of right ankle pain. Swelling and bruising of the right ankle was noted, and a fracture of the distal right fibula was diagnosed by radiography. This fracture was attributed to his fall prior to admission and was likely not found at the ACH due to his altered sensation and other medical challenges at the time. An orthopedic physician placed a hard cast, and the patient was made non-weight-bearing of the RLE for 6 weeks. To protect the fracture, the patient returned to requiring the use of a mechanical lift for out-of-bed transfers. Therefore, compensations, such as using slide board transfers, were initiated early on as it was known that the patient would be non-weight-bearing for at least 6 weeks. The patient was also educated on proper techniques for sit-to-stand and stand-pivot transfers so that when he was able to weight-bear through his RLE, it would not be a new concept.

**Table 2:** Physical therapy lower extremity assessments.

Assessment		T0^a^		T1^b^		T2^c^
**BLE^d^ strength**						
	ROM^e^	—^f^	WNL^g^		WNL	
**RLE^h^ strength**						
	Hip flexion	—	3/5		4–/5	
	Hip^i^	—	2+/5		3/5	
	Knee^j^	—	4/5		5/5	
	Ankle dorsiflexion	—	3/5		—; CAM^k^ boot	
**LLE^l^ strength**						
	Hip flexion	—	3/5		3+/5	
	Hip^i^	—	2/5		3/5	
	Knee^j^	—	3/5		4+/5	
	Ankle dorsiflexion	—	3–/5		3/5	
**Function and mobility^m^**						
	Out-of-bed transfers	D^m^	MaxA^n^; D after fracture was found		S^o^	
	Sitting balance	MinA^p^	CG^q^		I^r^	
	Sit-to-stand	D	D		S with RW^s^	
	Ambulatory transfers	UA^t^	UA		S with RW	
	Ambulation	UA	UA		300 feet; CG with RW	
	Stairs	UA	UA		Able to clear six 4-inch stairs; CG with bilateral railing	
**AM-PAC^u^ PT^v^**		—		10 points; 77% impairment		20 points; 36% impairment

^a^T0: acute care hospital (ACH) PT admission assessment, November 25, 2020; patient LE strength at the ACH was not formerly assessed or not available at the time of writing. Function and mobility assessments were available.

^b^T1: long-term acute care hospital (LTACH) PT admission assessment, November 28, 2020.

^c^T2: LTACH PT discharge assessment, January 20, 2021.

^d^BLE: bilateral lower extremities.

^e^ROM: range of motion.

^f^Not assessed at this time.

^g^WNL: within normal limits.

^h^RLE: right lower extremity.

^i^Measurement of both abduction and adduction.

^j^Measurement of both flexion and extension.

^k^CAM: controlled ankle motion.

^l^Measurements based on the modified functional independence measure score [35].

^m^D: dependent.

^n^MaxA: maximal assistance required.

^o^S: supervision required.

^p^MinA: minimal assistance required.

^q^CG: contact guard assistance required.

^r^I: independent.

^s^RW: rolling-walker assistive device required.

^t^UA: unable to perform.

^u^AM-PAC: activity measure for postacute care.

^v^PT: physical therapy.

**Table 3:** Modified functional independence measure definitions and criteria.

Descriptor	Definition
Unable (UA)	The subject/patient is unable to perform.
Dependent (D)	Dependent mobility; the subject/patient providing less than 25% of the work.
Maximal assistance (MaxA)	The subject/patient performs 25%-49% of the work.
Moderate assistance (ModA)	The subject/patient performs 50%-74% of the work.
Minimal assistance (MinA)	The subject/patient performs 75%-100% of the work.
Contact guard (CG)	The subject/patient requires light hands-on assistance for balance, but no physical lifting is required.
Close supervision (CS)	The subject/patient requires the therapist to be close by in case the patient experiences a loss of balance, but does not need physical or hands-on assistance.
Supervision (S)	During supervision, the therapist provides supervision at more than an arm’s length away.
Distant supervision (DS)	This is intermittent supervision. The therapist does not have to be in the room.
Modified independence (ModI)	The subject/patient is independent *with* the use of adaptive devices, techniques, or increased time.
Independent (I)	The subject/patient is independent *without* the use of adaptive devices, techniques, or increased time.

## Methods

### Therapy Details

During his inpatient stay, Monday through Friday, the patient participated in 5-6, 30-minute-long treatment blocks each day. These blocks consisted of a combination of individual PT and OT sessions, an OT/PT cotreatment session, LE and UE exercise group sessions, or a fine motor coordination group session focused on sensation and coordination. On Saturdays, he alternated weekly between a 30-minute LE or UE exercise group; treatment sessions were not conducted on Sundays.

During his early PT sessions, the patient worked on antigravity supine LE therapeutic exercises. He required skilled PT intervention to ensure the exercises were being performed properly and he was working at the appropriate workload. For example, the patient required assistance to perform hip flexion exercises in a side-lying position as, when he began to fatigue, he would compensate and recruit other muscles to facilitate the motion. As such, he required cues to stop and rest so that form was not compromised and he did not overfatigue the muscle. The patient also worked on wheelchair mobility and both static and dynamic sitting balance (ie, anterior weight shifting and reaching outside the base of support to prepare for transfers).

In early OT sessions, the patient worked on bed-level lower body ADLs, UE exercises seated on a mat, cognitive tasks addressing short-term memory, and hand-strengthening activities. Additionally, the patient began participating in OT convergence training exercises to address his convergence insufficiencies. Within a week of starting convergence training, the patient started to report improvements to the blurry vision and increased ability to read novels at his leisure.

UE exercises focused on using lighter resistance weights that the patient could tolerate for 10 repetitions without compromising body mechanics; the weight was gradually increased per the patient’s tolerance. Due to shoulder weakness, passive ROM exercises were initiated to the joint end ROM to preserve joint integrity. In addition to performing daily OT tasks, which included self-ROM and hand-strengthening tasks with putty (eventually increasing to graded grippers), the patient was independent with a gentle bed-level exercise program.

The patient was educated on energy conservation during ADL performance, including taking rest breaks, utilizing adaptive equipment to improve independence without compromising functional activity tolerance, and preserving the ability to persist through daily therapies [16]. In his early cotreatment sessions, PT and OT worked together to assist the patient with bed mobility and basic slide board transfers to and from his wheelchair and bed. As the patient progressed, OT/PT cotreatments worked on slide board transfers to and from the car, tub, and commode.

On January 7, 2021, 18 days prior to discharge, the patient started progressive LE strengthening, sit-to-stand transfers, and ambulation during individual PT sessions. The individual OT sessions were able to focus on progressing UE therapeutic exercises, dressing at a wheelchair level, and bathing in the shower using a shower chair.

OT/PT cotreatment sessions also advanced the patient from standing to using a rolling-walker (RW) with a right sling attachment to support his RLE. This allowed him to weight-bear through the right knee to provide more stability while standing and ambulating and adhere to his weight-bearing restrictions. As the patient progressed, he no longer required cotreatment for standing and ambulation and was able to perform these activities with the assistance of 1 person. This allowed for an additional individual therapy session each day.

On January 15, 2021, 44 days after being placed in the hard cast, the patient was cleared for weight-bearing as tolerated through his RLE using a controlled ankle motion (CAM) walking boot and RW. To ensure he did not have a significant increase in pain with weight-bearing, the patient’s ambulation distance was gradually increased. As he began tolerating ambulating further distances with the RW, he began climbing stairs with assistance.

During his PT LE exercise group session, the patient performed seated LE exercises, including hip flexion, hip abduction/adduction, knee extension, and ankle ROM. To help his progression, ankle weights were added, as appropriate, per therapist discretion and the patient’s tolerance. The patient was also able to utilize a recumbent cross-trainer using his LLE and BUEs during these sessions. Once cleared for weight-bearing with the CAM boot, he was able to effectively use all extremities on the cross-trainer. During his OT UE exercise group session, the patient performed seated UE exercises, starting without weights and gradually worked up to 4 lb in shoulder weights and 6 lb in elbow weights. In the fine motor coordination group session, the patient began with hand-strengthening and larger fine motor coordination tasks; he gradually transitioned to smaller tasks, including putting small objects (approximately 0.5 inches) together and pulling them apart.

### Ethics Approval and Consent to Participate

This case report was written in compliance with our institutional privacy policy, the Health Insurance Portability and Accountability Act (HIPAA) policy, and the standards set by the Declaration of Helsinki. Institutional review board approval was not required by institutional policy as the report only describes 1 patient; the need for approval was therefore waived.

### Consent for Publication

The patient described here gave his written permission for the authors to access his personal information and for his information to be used in writing and publishing this case report.

## Results

### Discharge Assessment

The patient’s function was re-evaluated immediately prior to LTACH discharge. The patient’s PT discharge assessment indicated that his LE coordination and ROM were now WNL and that his LE strength was as follows:

- RLE: hip flexion 4–/5, hip abduction/adduction 3/5, and knee flexion/extension 5/5
- LLE: hip flexion 3+/5, hip abduction/adduction 3/5, knee flexion 4+/5, knee extension 4–/5, and ankle dorsiflexion 3/5

The patient’s right ankle was unable to be assessed as it was still in a CAM walking boot and not cleared for ROM assessments (Table 2).

The patient continued to endorse diminished sensation in his bilateral lower legs and feet, but his sensation to light touch had returned. Proprioception was WNL in his BLEs. Functionally, the patient was performing sit-to-stand and ambulatory transfers with an RW with supervision. By discharge, he had regained ambulation and was able to ambulate 300 feet with an RW and CG utilizing the CAM walking boot. Further, the patient was able to negotiate six 4-inch stairs with bilateral railings, step-to-pattern, and a CG. The patient scored a 20 on the AM-PAC at discharge, indicating a 36% impairment; this was a 41% improvement from admission (Table 2).

The patient’s OT discharge assessment indicated that his BUE strength, coordination, and ROM were now WNL (Table 1). His grip strength was 52 lb on the right and 70 lb on the left, a 37 and 49 lb increase, respectively. Although the patient continued to demonstrate diminished sensation to light touch in BUEs, his proprioception remained intact. With regard to ADLs, the patient could now perform upper body bathing/dressing and lower body bathing with distant supervision; lower body dressing sitting upright in the wheelchair could be performed with a CG. He was also able to complete ambulatory transfers to the commode and transfer to a tub bench with supervision.

The patient’s visual impairments were completely resolved upon discharge. With residual impairments to his short-term memory still present, the patient scored a 27/30 on the SLUMS examination, a 2-point improvement. He also scored a 20 on the AM-PAC at discharge, indicating a 35% impairment; this was a 15% improvement from admission (Table 1).

On January 21, 2021, 56 days after LTACH admission, the patient was discharged home, ambulatory with improved strength, self-care, and cognition.

## Discussion

### Principal Findings

Here we described the presentation and rehabilitation regimen of a patient diagnosed with COVID-19-associated GBS. During a telehealth appointment following a COVID-19 diagnosis, the patient fell. Being unable to stand up due to weakness in his LEs, the medical professional advised the patient to call for emergency medical services. He was then brought to a local regional ACH. There he was diagnosed and treated for GBS. Requiring functional rehabilitation, the patient was transferred to an independent rehabilitation-focused LTACH, where OT and PT regimens led to objective improvements in both UE and LE strength. Fine motor control and coordination were also markedly improved, as evidenced by the patient’s ability to open containers, write, and self-feed. The patient’s functional mobility improved from being dependent and unable to ambulate or perform transfers to ambulation with a CG and transfers with supervision. This led to functional improvements, independence with ADLs, improved AM-PAC scores for both the mobility and ADL sections, and a safe discharge home.

PT and OT interventions were structured to optimize the patient’s independence at each stage of rehabilitation. Although the patient was challenged throughout the week, therapists were careful to not overfatigue the patient so as to avoid potential delays in his recovery and to allow for rest on weekends. When resuming therapy on Mondays, the patient had objectively notable increases in both his UE and his LE strength. It is important to provide patients with adequate rest in order to maximize functional recovery.

### Strengths and Limitations

It is important to acknowledge the strengths and limitations of this report. This report was strengthened by the use of objective measures and standardized assessments to demonstrate the improvements this patient made at an LTACH level of care. Although GBS is an exceedingly rare disease, it is relatively common for patients with GBS to be treated at our facility each year. Although this report is based off 1 patient’s case, our facility has admitted and treated 5 patients with GBS related to COVID-19 at the time of writing. A limitation of this particular case is the absence of some diagnostic tests typically reported for GBS, namely cerebrospinal fluid analysis and electrophysiological nerve conduction studies. Being an independent LTACH, the records we were able to obtain from the ACH, where the patient was initially diagnosed and treated, were incomplete. However, given the abundance of literature already describing the diagnosis and treatment of COVID-19-related GBS, we reported what was available to us and focused on reporting the rehabilitation regimen used to treat this patient for COVID-19-related GBS. Additionally, nerve conduction studies could have been used to monitor the patient’s recovery and rehabilitation. However, as this is not a common practice at our institution and the patient’s recovery and rehabilitation were in line with expectations, follow-up nerve conduction studies were not conducted.

A challenge in this patient’s recovery was that his ankle fracture left him non-weight-bearing for much of his rehabilitation. Although it is not typical for patients with GBS to also have weight-bearing restrictions, we thought reporting this unique case worthwhile as no 2 patients will require the same rehabilitation regimen. In fact, it is often the case that patients will arrive for rehabilitation with not 1 but multiple diagnoses that will influence their treatment plan. Being no exception, the patient’s non-weight-bearing status influenced his treatment course and added challenges in his functional mobility progress that were addressed with an individualized therapeutic approach. These goals were centered on the patient becoming as mobile as possible, given his weight-bearing restrictions, while being careful to not overfatigue his muscles and creating additional problems. This patient’s rehabilitation may have been complicated by this fracture and non-weight-bearing restrictions, but the patient made significant improvements in his time at this LTACH and recovered, as we would expect a patient with GBS to recover.

### Patient’s Perspective

Following the patient’s discharge, he was asked to give his thoughts about his time in rehabilitation. From the first day the patient started therapy, he was motivated and had a positive attitude. The patient admits, “I did have some mental difficulties early on absorbing all that was happening to me.” This was never shown outwardly, however, and the patient quickly moved away from this mindset. The patient states, “I was somewhat traumatized from the 8 days in the [acute care] hospital all alone, but once I realized that everyone at Gaylord was looking out for me, I was able to relax and just focus on getting better. I worked hard to maintain a positive attitude and to talk with other patients and staff to get encouragement and strength from them so I could reflect on it each night, which was the most difficult time for me.” This mentality helped the patient to overcome the obstacles he faced during rehabilitation and make a remarkable recovery.

### Conclusion

At the time of writing, this is the first report to the best of our knowledge to demonstrate the standard of care and strategies used in an independent LTACH setting to successfully rehabilitate and discharge a patient diagnosed with GBS following COVID-19 infection and the second report to describe these methods overall [33]. This successful rehabilitation was accomplished through intensive PT and OT regimens targeted at patient-specific deficits. This case report demonstrates how therapy interventions are effective in maximizing the functional potential of patients with COVID-19-associated GBS.

## Abbreviations

ACH: acute care hospital
ADL: activity of daily living
AIDP: acute inflammatory demyelinating polyradiculoneuropathy
AM-PAC: activity measure for postacute care
BUE: bilateral upper extremity
CAM: controlled ankle motion
GBS: Guillain-Barré syndrome
IVIG: intravenous immunoglobulin
LE: lower extremity
LLE: left lower extremity
LTACH: long-term acute care hospital
LUE: left upper extremity
MERS: Middle East respiratory syndrome
MMT: manual muscle testing
MRI: magnetic resonance imaging
OT: occupational therapy
PCR: polymerase chain reaction
PT: physical therapy
RLE: right lower extremity
RUE: right upper extremity
SLUMS: St. Louis University Mental States
UE: upper extremity
WNL: within normal limits

## References

[1] Sauer L What Is Coronavirus?.

[2] World Health Organization WHO Coronavirus (COVID-19) Dashboard.

[3] Willison HJ, Jacobs BC, van Doorn PA (2016). Guillain-Barré syndrome. Lancet.

[4] van den Berg B, Walgaard C, Drenthen J, Fokke C, Jacobs BC, van Doorn PA (2014). Guillain-Barré syndrome: pathogenesis, diagnosis, treatment and prognosis. Nat Rev Neurol.

[5] Ellul MA, Benjamin L, Singh B, Lant S, Michael BD, Easton A, Kneen R, Defres S, Sejvar J, Solomon T (2020). Neurological associations of COVID-19. Lancet Neurol.

[6] Rahimi K (2020). Guillain-Barre syndrome during COVID-19 pandemic: an overview of the reports. Neurol Sci.

[7] Pergolizzi JV, Raffa R, Varrassi G, Magnusson P, LeQuang J, Paladini A, Taylor R, Wollmuth C, Breve F, Chopra M, Nalamasu R, Christo P (2021). Potential neurological manifestations of COVID-19: a narrative review. Postgrad Med.

[8] Liu Y, Sawalha A, Lu Q (2020). COVID-19 and autoimmune diseases. Curr Opin Rheumatol.

[9] Dotan A, Muller S, Kanduc D, David P, Halpert G, Shoenfeld Y (2021). The SARS-CoV-2 as an instrumental trigger of autoimmunity. Autoimmun Rev.

[10] Sriwastava S, Kataria S, Tandon M, Patel J, Patel R, Jowkar A, Daimee M, Bernitsas E, Jaiswal P, Lisak RP (2021). Guillain Barré syndrome and its variants as a manifestation of COVID-19: a systematic review of case reports and case series. J Neurol Sci.

[11] Abdullahi A, Candan SA, Soysal Tomruk M, Elibol N, Dada O, Truijen S, Saeys W (2020). Is Guillain-Barré syndrome associated with COVID-19 infection? a systemic review of the evidence. Front Neurol.

[12] Centers for Disease Control and Prevention Campylobacter (Campylobacteriosis): Guillain-Barré Syndrome.

[13] Toscano G, Palmerini F, Ravaglia S, Ruiz L, Invernizzi P, Cuzzoni MG, Franciotta D, Baldanti F, Daturi R, Postorino P, Cavallini A, Micieli G (2020). Guillain–Barré syndrome associated with SARS-CoV-2. N Engl J Med.

[14] Mayo Clinic Guillain-Barre Syndrome: Symptoms and Causes.

[15] Novak P, Šmid S, Vidmar G (2017). Rehabilitation of Guillain-Barré syndrome patients: an observational study. Int J Rehabil Res.

[16] GBS/CIDP Foundation International Foundation Publications: Guidelines for Physical and Occupational Therapy.

[17] Korem S, Gandhi H, Dayag DB (2020). Guillain-Barré syndrome associated with COVID-19 disease. BMJ Case Rep.

[18] Sedaghat Z, Karimi N (2020). Guillain Barre syndrome associated with COVID-19 infection: a case report. J Clin Neurosci.

[19] Miller A, Burke R, Gest A, Ramanandi VH (2020). Rehabilitation of post-COVID GBS patient: acute care and functional recovery. Res Pedtr Neonatol.

[20] Manganotti P, Bellavita G, Tommasini V, D Acunto L, Fabris M, Cecotti L, Furlanis G, Sartori A, Bonzi L, Buoite Stella A, Pesavento V (2021). Cerebrospinal fluid and serum interleukins 6 and 8 during the acute and recovery phase in COVID-19 neuropathy patients. J Med Virol.

[21] Singh R, Shiza ST, Saadat R, Dawe M, Rehman U (2021). Association of Guillain-Barre syndrome with COVID-19: a case report and literature review. Cureus.

[22] Avenali M, Martinelli D, Todisco M, Canavero I, Valentino F, Micieli G, Alfonsi E, Tassorelli C, Cosentino G (2021). Clinical and electrophysiological outcome measures of patients with post-infectious neurological syndromes related to COVID-19 treated with intensive neurorehabilitation. Front Neurol.

[23] Türk Börü Ü, Köseoğlu Toksoy C, Bölük C, Demirbaş H, Yılmaz AÇ (2021). A case of Guillain-Barré syndrome related to COVID-19 infection. Int J Neurosci.

[24] Gale A, Sabaretnam S, Lewinsohn A (2020). Guillain-Barré syndrome and COVID-19: association or coincidence. BMJ Case Rep.

[25] Garnero M, Del Sette M, Assini A, Beronio A, Capello E, Cabona C, Reni L, Serrati C, Bandini F, Granata A, Pesce G, Mancardi GL, Uccelli A, Schenone A, Benedetti L (2020). COVID-19-related and not related Guillain-Barré syndromes share the same management pitfalls during lock down: the experience of Liguria region in Italy. J Neurol Sci.

[26] Manganotti P, Bellavita G, D'Acunto L, Tommasini V, Fabris M, Sartori A, Bonzi L, Buoite Stella A, Pesavento V (2021). Clinical neurophysiology and cerebrospinal liquor analysis to detect Guillain-Barré syndrome and polyneuritis cranialis in COVID-19 patients: a case series. J Med Virol.

[27] Khalifa M, Zakaria F, Ragab Y, Saad A, Bamaga A, Emad Y, Rasker JJ (2020). Guillain-Barré syndrome associated with severe acute respiratory syndrome coronavirus 2 detection and coronavirus disease 2019 in a child. J Pediatric Infect Dis Soc.

[28] Tiet MY, AlShaikh N (2020). Guillain-Barré syndrome associated with COVID-19 infection: a case from the UK. BMJ Case Rep.

[29] Bracaglia M, Naldi I, Govoni A, Brillanti Ventura D, De Massis P (2020). Acute inflammatory demyelinating polyneuritis in association with an asymptomatic infection by SARS-CoV-2. J Neurol.

[30] Tatu L, Nono S, Grácio S, Koçer S (2021). Guillain-Barré syndrome in the COVID-19 era: another occasional cluster?. J Neurol.

[31] Assini A, Benedetti L, Di Maio S, Schirinzi E, Del Sette M (2020). New clinical manifestation of COVID-19 related Guillain-Barrè syndrome highly responsive to intravenous immunoglobulins: two Italian cases. Neurol Sci.

[32] Su XW, Palka SV, Rao RR, Chen FS, Brackney CR, Cambi F (2020). SARS-CoV-2-associated Guillain-Barré syndrome with dysautonomia. Muscle Nerve.

[33] Colonna S, Sciumé L, Giarda F, Innocenti A, Beretta G, Dalla Costa D (2020). Case report: postacute rehabilitation of Guillain-Barré syndrome and cerebral vasculitis-like pattern accompanied by Sars-CoV-2 infection. Front Neurol.

[34] Shirley Ryan AbilityLab Manual Muscle Test.

[35] Physiopedia Functional Independence Measure (FIM).

[36] Shirley Ryan AbilityLab Activities-Specific Balance Confidence Scale.

[37] Tariq SH, Tumosa N, Chibnall JT, Perry MH, Morley JE (2006). Comparison of the Saint Louis University mental status examination and the mini-mental state examination for detecting dementia and mild neurocognitive disorder: a pilot study. Am J Geriatr Psychiatry.

[38] National Institute of Neurological Disorders and Stroke Miller Fisher Syndrome Information Page: What Research Is Being Done?.

